# Association of the plasma xanthine oxidoreductase activity with the metabolic parameters and vascular complications in patients with type 2 diabetes

**DOI:** 10.1038/s41598-021-83234-9

**Published:** 2021-02-12

**Authors:** Tomoko Okuyama, Jun Shirakawa, Takashi Nakamura, Takayo Murase, Daisuke Miyashita, Ryota Inoue, Mayu Kyohara, Yu Togashi, Yasuo Terauchi

**Affiliations:** 1 Department of Endocrinology and Metabolism, Graduate School of Medicine, Yokohama City University, Yokohama, Japan; 2 Laboratory and Diabetes and Metabolic Disorders, Institute for Molecular and Cellular Regulation (IMCR), Gunma University, 3-39-15 Showa-machi, Maebashi, 371-8512 Japan; 3 Mie Research Laboratories, Sanwa Kagaku Kenkyusho Co., Ltd., Inabe, Mie Japan

**Keywords:** Biomarkers, Endocrinology

## Abstract

Xanthine oxidoreductase (XOR) catalyzes the oxidation of hypoxanthine to xanthine, and of xanthine to uric acid. XOR also enhances the production of reactive oxygen species and causes endothelial dysfunction. In this study, we evaluated the association of XOR and its substrate with the vascular complications in 94 Japanese inpatients with type 2 diabetes (T2DM). The plasma XOR activity and plasma xanthine levels were positively correlated with the body mass index, aspartate aminotransferase (AST), alanine aminotransferase (ALT), γ-GTP, fasting plasma insulin, and the homeostasis model of assessment of insulin resistance (HOMA-IR), and negatively correlated with the high density lipoprotein cholesterol. The plasma XOR activity also showed a positive correlation with the serum triglyceride. Multivariate analyses identified AST, ALT, fasting plasma insulin and HOMA-IR as being independently associated with the plasma XOR activity. The plasma XOR activity negatively correlated with the duration of diabetes, and positively correlated with the coefficient of variation of the R-R interval and sensory nerve conduction velocity. Furthermore, the plasma XOR activity was significantly decreased in patients with coronary artery disease. Thus, the plasma XOR activity might be a surrogate marker for the development of vascular complications, as well as liver dysfunction and insulin resistance, in T2DM.

Trial registration: This study is registered at the UMIN Clinical Trials Registry (UMIN000029970; https://www.umin.ac.jp/ctr/index-j.htm). The study was conducted from Nov 15, 2017.

## Introduction

Increasing evidence has been accumulated to show an association between oxidative stress and the pathogenesis of diabetes, as well as obesity, cardiovascular disease, heart failure, cancer, hypertension, atherosclerosis, and inflammatory disease^1,2^. Chronic hyperglycemia enhances the production of reactive oxygen species (ROS) and is known to induce oxidative stress, contributing to the development of insulin resistance, β-cell dysfunction, and vascular complications^3^.

Xanthine oxidoreductase (XOR), which includes both xanthine oxidase (XO) and xanthine dehydrogenase (XDH), is a rate-limiting enzyme in purine catabolism and production of uric acid. In the purine catabolic pathway, XOR oxidizes hypoxanthine to xanthine, and xanthine to uric acid^4^. Since a considerable amount of H_2_O_2_ and O_2_^−^ is generated during the catalytic activity of XOR, XOR is considered to play a central role in the production of ROS and induction of vascular endothelial dysfunction in patients with type 2 diabetes^5^. Under the condition of chronic hyperglycemia, production of ROS is enhanced via multiple pathways, including such as the activated polyol pathway, hexamine pathway, protein kinase C (PKC) pathway, O_2_^−^ production from mitochondria, accumulation of advanced glycation end products (AGE), and activation of XOR^3^. On the other hand, in the cardiovascular system, activated NADPH oxidase via angiotensin II stimulation, O_2_^−^ production from the myocardial mitochondria, and activated XOR are responsible for the production of ROS during cardiovascular remodeling^6^.

Increased XOR activity has been reported to be observed in patients with coronary artery disease^7^, heart failure and obesity^8^, and also both in patients with type 1 and 2 diabetes mellitus (hereinafter simply, diabetes)^9,10^. XOR activity has also been reported to be associated with the risk of cardiovascular events^11–13^. Both insulin resistance and liver dysfunction have been reported to be correlated with the plasma XOR activity in the general population^14^. Several studies have reported an association of the plasma XOR activity with the HbA1c and risk of development of peripheral neuropathy in patients with diabetes^10,15^. However, the association of the XOR activity with the metabolic parameters/risk of vascular complications in patients with type 2 diabetes remains obscure. Moreover, little is known about the significance of the plasma levels of purines and the XOR activity in patients with type 2 diabetes. Therefore, we hypothesized that the plasma XOR activity and purine levels might be useful as surrogate markers for diabetic vascular complications.

In this study, we investigated the plasma XOR activity, xanthine and hypoxanthine levels in patients with type 2 diabetes to clarify their associations with the metabolic parameters and vascular complications in type 2 diabetes.

## Methods

### Study participants

We enrolled 94 Japanese patients with type 2 diabetes who were not receiving treatment for hyperuricemia. Patients who were pregnant, had severe renal dysfunction with an estimated glomerular filtration rate (eGFR) of < 30 ml/min/m^2^, severe liver dysfunction, ketosis or infection, or any cancer were also excluded. For this study, patients with cardiovascular disease (CVD) were defined as those with coronary artery disease, ischemic cerebrovascular disease, or atherosclerotic peripheral artery disease (PAD). A patient was defined as having PAD when any of the following criteria was met: aorto-femoral bypass surgery, limb bypass surgery, percutaneous transluminal angioplasty revascularization of the iliac, or infra-inguinal arteries; limb or foot amputation for arterial vascular disease; intermittent claudication and one or more of either an ankle brachial index (ABI) of less than 0.9 or a peripheral arterial stenosis (≥ 50%) documented by angiography or duplex ultrasound; carotid revascularization or asymptomatic carotid arterial stenosis of at least 50% diagnosed by duplex ultrasound or angiography^16,17^. All the patients were Japanese patients who were hospitalized at the Yokohama City University Hospital, Yokohama, Japan. This study was carried out from April 2017 to March 2019, with the approval of the institutional ethics committee (approval number: B170900049, institutional ethical committee of Yokohama City University), and in accordance with the Declaration of Helsinki. All patients provided informed consent and signed informed consent forms.

### Blood sampling and measurement of the plasma XOR activity, xanthine, and hypoxanthine levels

Blood samples were collected from the patients in the fasting condition early in the morning, and then centrifuged at 3000 rpm for 10 min at 4 °C within 8 h after blood collection to avoid the leak of hypoxanthine and xanthine from erythrocytes into the plasma^18^. The supernatant plasma samples were maintained at -80 °C until the assay. Plasma XOR activity was determined as previously described^19^. In brief, plasma sample was purified using a Sephadex G25 column, and 100 µL aliquots of the eluate was then mixed with [^13^C_2_, ^15^N_2_]-xanthine as the substrate and NAD^+^ and [^13^C_3_, ^15^N_3_]-uric acid as the internal standard in Tris buffer (pH 8.5). Each of the mixtures was incubated at 37 °C for 90 min, quenched by methanol, and centrifuged at 2000 × g for 15 min at 4 °C. The supernatants transferred to new tubes were evaporated, reconstituted with 150 µL distilled water, and filtered through an ultrafiltration membrane before undergoing a liquid chromatography-triple quadrupole mass spectrometry (LC/TQMS) analysis using the Nano Space SI-2 LC system (Shiseido, Ltd., Tokyo, Japan) and a TSQ-Quantum TQM spectrometer (Thermo Fisher Scientific, Bremen, Germany) equipped with an electrospray ionization source. The amount of [^13^C_2_, ^15^N_2_]-uric acid produced was quantified using the calibration curve, with the XOR activity expressed as [^13^C_2_, ^15^N_2_]-uric acid in pmol/h/mL plasma. The lower limit of detection was 6.67 pmol/h/mL plasma, and intra- and inter-assay coefficients of variation were 6.5% and 9.1%, respectively. Plasma hypoxanthine and xanthine were also measured as previously reported^18,19^. In brief, plasma samples were added into methanol containing [^13^C_2_, ^15^N_2_] xanthine and [^13^C_3_, ^15^ N] hypoxanthine as internal standard and were centrifuged by 3000 × g at 4 °C for 15 min. The supernatant was diluted with distilled water, and concentrations of hypoxanthine and xanthine were measured using LC/TQMS (Nexera SCIEX QTRAP 4500, SHIMADZU, Japan).

### Statistical analysis

Statistical analyses were performed using SPSS statics 19 (IBM). Variables are presented as means ± standard deviation, medians (interquartile range) or number (%). Comparisons between two groups were carried out by Student’s t-test and the Mann–Whitney U test for continuous variables, and by the chi-square test for categorical variables. Statistical calculations for significant differences were carried out using the Wilcoxon signed-rank test, Wilcoxon rank sum test and Spearman’s rank correlation coefficients. Multivariate regression analyses were carried out to identify independent associations between the plasma XOR activity and variables, and the standardized regression coefficient (β) and percentage of variance for the selected independent predictors explained (R^2^). Statistical significance was set at *P* < 0.05.

### Ethics approval

This study was approved by the institutional ethics committee at each participating hospital and conducted in accordance with the Declaration of Helsinki.

### Consent to participate

All patients provided written informed consent prior to participation.

## Results

### Clinical characteristics of the participants

The clinical characteristics of the 94 participants (male; 59, female; 35) are shown in Table 1. The age was 64 ± 12 years, the BMI was 26.2 ± 7.1 kg/m^2^, and the HbA1c was 8.8% (7.9–10.1). The duration of diabetes was 10 ± 11 years. The plasma XOR activity was 67.7 pmol/h/mL (31.1–184). The plasma concentrations of xanthine and hypoxanthine were 0.71 µM (0.60–0.86) and 3.6 µM (2.8–5.3), respectively. There were no significant differences in the age, plasma XOR activity, plasma xanthine level, plasma hypoxanthine level or serum uric acid level between the male and female patients (see Supplementary Fig. 1). Serum uric acid level and plasma XOR activity have been reported to be higher in men than in women in the general population. Under the diabetic condition, the serum uric acid level is expected to be influenced by hyperfiltration because of increased eGFR and urinary sugar-associated excretion of urate. In addition to the altered circulating uric acid metabolism, the multifactorial effects of factors such as hyperglycemia, obesity, liver dysfunction, dyslipidemia, and endothelial dysfunction possibly abolished the sex difference in the plasma XOR activity in the patients in the current study. In another study, the plasma XOR activity was reported to be higher in patients with type 2 diabetes than in the general population^14^, consistent with the elevated plasma XOR activity observed in the patients with diabetes in this study.

**Table 1:** Clinical characteristics of the study patients.

Number (male/female)	94 (59:35)
Age (years)	64 ± 12
Duration of diabetes (years)	10 ± 11
BMI (kg/m^2^)	26.2 ± 7.1
Waist circumference (cm)	97.3 ± 15.0
**Disease**	
Hypertension	51 (54.3)
Cardiovascular disease	20 (21.2)
**Medications**	
Insulin	27 (28.7)
GLP-1 receptor agonist	7 (7.4)
Metformin	32 (34.0)
DPP-4 inhibitor	49 (52.1)
SGLT2 inhibitor	16 (17.0)
Thiazolidine	9 (9.6)
Sulfonylurea	10 (10.6)
Glinide	7 (7.4)
α-Glucosidase inhibitor	12 (12.8)
HbA1c (%)	8.8 (7.9–10.1)
Glycated albumin (%)	22.0 (18.5–26.7)
Fasting plasma glucose (mg/dL)	147 (129–185)
Fasting IRI (μIU/mL)	8.8 (4.3–13.9)
Fasting CPR (ng/mL)	2.4 (1.4–3.0)
HOMA-IR	3.3 (1.7–5.1)
eGFR (mL/min/1.73 m^2^)	75 (63–91)
ACR (mg/g · Cr)	10.8 (4.6–43.9)
UA (mg/dL)	5.4 (4.0–6.2)
T-Cho (mg/dL)	193 (169–214)
HDL-C (mg/dL)	49 (40–57)
LDL-C (mg/dL)	113 (97–136)
TG (mg/dL)	134 (95–185)
AST (IU/L)	21 (17–31)
ALT (IU/L)	21 (14–38)
γ-GTP (IU/L)	32 (20–48)
Fib-4 index	1.4 (0.9–1.9)
Urinary CPR (μg/day)	78 (46–110)
Plasma XOR activity (pmol/h/mL)	67.7 (31.1–184)
Xanthine (μM)	0.71 (0.60–0.86)
Hypoxanthine (μM)	3.6 (2.8–5.3)
CVR-R (%)	2.0 (1.3–2.7)
MCV (m/s)	42.2 (38.9–45.6)
SCV (m/s)	42.4 (37.2–47.6)
Diabetic retinopathy	25 (26.6)
ABI	1.13 (1.07–1.19)
baPWV (cm/sec)	1645 (1403–1912)

Data are presented as means ± standard deviation, medians (interquartile range) or number (%). *BMI* body mass index, *HbA1c* glycosylated haemoglobin, *IRI* immunoreactive insulin, *CPR* C-peptide immunoreactivity, *HOMA-IR* homeostasis model assessment of insulin resistance, *eGFR* estimated glomerular filtration rate, *ACR* urinary albumin to creatinine ratio, *UA* serum uric acid level, *T-Chol* serum total cholesterol level, *HDL-C* serum high-density lipoprotein cholesterol level, *LDL-C* serum low density lipoprotein cholesterol level, *TG* serum triglyceride level, *AST* serum aspartate aminotransferase level, *ALT* serum alanine aminotransferase level, *γ-GTP* serum γ-glutamyl transpeptidase level, *plasma XOR activity* plasma xanthine oxidoreductase activity level, *CVR-R* coefficient of variation of the R-R interval, *MCV* motor nerve conduction velocity (tibial nerve), *SCV* sensory nerve conduction velocity (peroneal nerve), *ABI* ankle brachial index, *baPWV* brachial-ankle pulse wave velocity.

**Figure 1:**
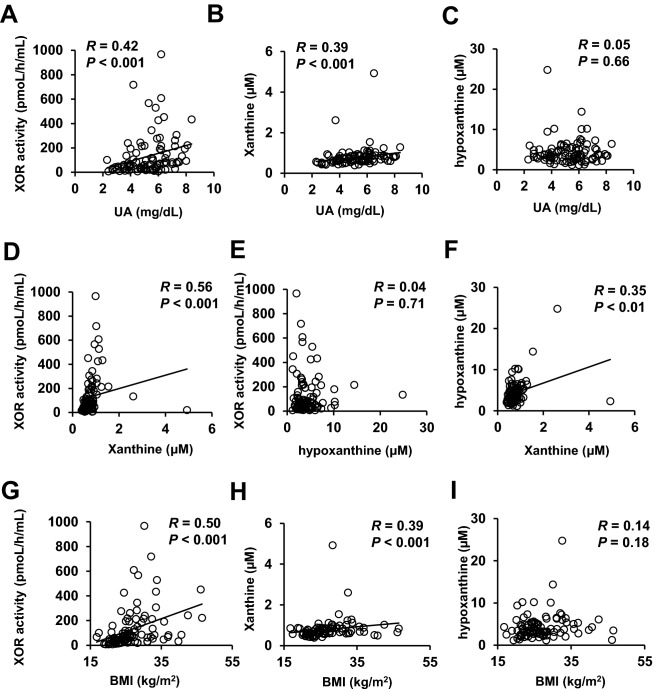
Correlations among the plasma XOR activity and plasma levels of xanthine and hypoxanthine. (**A**) Correlation between the plasma XOR activity (pmol/h/mL) and serum uric acid level. (**B**) Correlation between the plasma xanthine concentration (µM) and serum uric acid level (mg/dL). (**C**) Correlation between the plasma hypoxanthine concentration (µM) and serum uric acid level (mg/dL). (**D**) Correlation between the plasma XOR activity (pmol/h/mL) and plasma xanthine concentration (µM). (**E**) Correlation between the plasma XOR activity (pmol/h/mL) and plasma hypoxanthine concentration (µM). (**F**) Correlation between the plasma xanthine concentration (µM) and plasma hypoxanthine concentration (µM). (**G**) Correlation between the plasma XOR activity (pmol/h/mL) and BMI (kg/m^2^). (**H**) Correlation between the plasma xanthine concentration (µM) and BMI (kg/m^2^). (**I**) Correlation between the plasma hypoxanthine concentration (µM) and BMI (kg/m^2^).

### Correlations among the plasma XOR activity and the plasma xanthine, hypoxanthine and uric acid levels

First, we investigated the correlations among the plasma XOR activity, plasma xanthine, plasma hypoxanthine, and serum uric acid levels by simple regression analyses (Fig. 1). The plasma XOR activity was significantly and positively correlated with the plasma level of xanthine (R = 0.56, *P* < 0.001) and the serum uric acid level (R = 0.42, *P* < 0.001), but not with the plasma hypoxanthine level (R = 0.04, *P* = 0.71). In contrast, a positive correlation was observed between the plasma xanthine and hypoxanthine levels (R = 0.35, *P* < 0.01). Plasma xanthine level, but not the plasma hypoxanthine level, was also correlated with the serum uric acid level (R = 0.39, *P* < 0.001).

### Correlations between the plasma XOR activity and metabolic parameters

**Figure 2:**
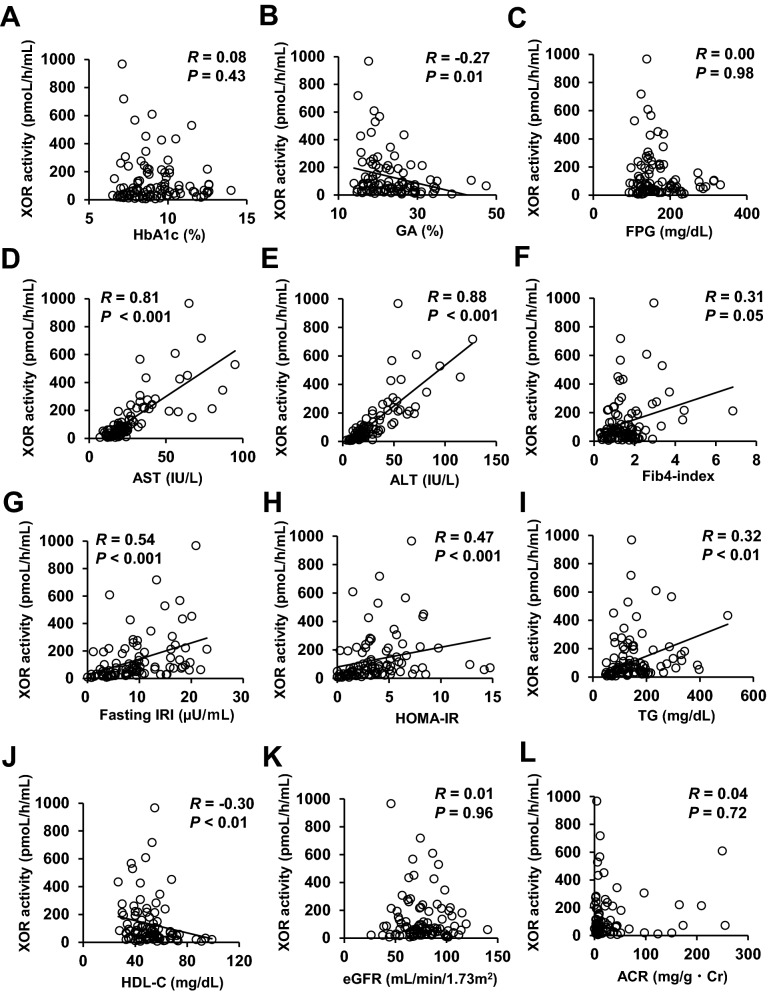
Correlations between the plasma XOR activity level and clinical parameters. Correlations between the plasma XOR activity (pmol/h/mL) and (**A**) HbA1c (%), (**B**) glycated albumin (%), (**C**) fasting plasma glucose level (mg/dL), serum levels of (**D**) AST (IU/L) and (**E**) ALT (IU/L), (**F**) Fib4-index, (**G**) fasting serum IRI (µU/mL), (**H**) HOMA-IR, (**I**) serum TG level (mg/dL), (**J**) HDL-C (mg/dL), (**K**) eGFR (ml/min/1.73m^2^), and (**L**) urinary albumin to creatinine ratio (ACR) (mg/g · Cr).

The correlations between the plasma XOR activity and the clinical parameters in the patients with type 2 diabetes were examined by simple regression analyses, and the results are shown in Fig. 2 and Supplementary Table 1. There was a significant positive correlation between the plasma XOR activity and the body mass index (BMI) (R = 0.50, *P* < 0.001). In addition, the plasma XOR activity showed correlations with parameters of insulin resistance, such as fasting immunoreactive insulin (IRI) (R = 0.54, *P* < 0.001), homeostasis model assessment of the index of insulin resistance (HOMA-IR) (R = 0.47, *P* < 0.001) and urinary C-peptide excretion during the day (R = 0.41, *P* = 0.01). In addition, the plasma XOR activity showed significant positive correlations with the AST (R = 0.81, *P* < 0.001), ALT (R = 0.88, *P* < 0.001), γ-GTP (R = 0.76, *P* < 0.001), and triglyceride (TG) (R = 0.32, *P* < 0.01), and negative correlations with the percent glycated albumin (R = − 0.27, *P* = 0.01) and HDL-C (R = − 0.30, *P* < 0.05); no significant correlation of the plasma XOR activity with the HbA1c (R = 0.08, *P* = 0.43) or the fasting plasma glucose level (R = 0.00, *P* = 0.98) was observed in the patients with type 2 diabetes. The plasma XOR activity showed no significant correlations with the parameters of diabetic nephropathy, eGFR, severity of albuminuria, and serum cystatin C either in the patients with type 2 diabetes.

### Correlations between the plasma xanthine concentrations and metabolic parameters

**Figure 3:**
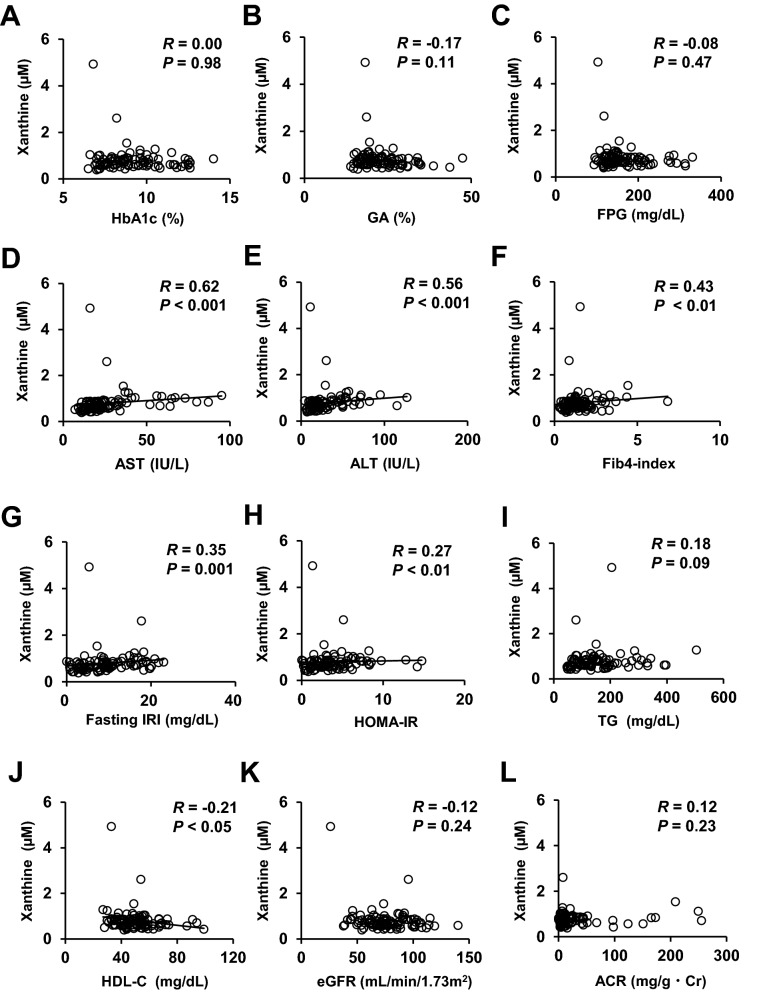
Correlations between the plasma xanthine level and clinical parameters. Correlations between the plasma concentration of xanthine (µM) and (**A**) HbA1c (%), (**B**) glycated albumin (%), (**C**) fasting plasma glucose level (mg/dL), serum levels of (**D**) AST (IU/L) and (**E**) ALT (IU/L), (**F**) Fib4-index, (**G**) fasting serum IRI (µU/mL), (**H**) HOMA-IR, (**I**) serum TG level (mg/dL), (**J**) HDL-C (mg/dL), (**K**) eGFR (ml/min/1.73m^2^), and (**L**) urinary albumin to creatinine ratio (ACR) (mg/g · Cr).

We also analyzed the correlations between the plasma xanthine levels and the clinical parameters in patients with type 2 diabetes by simple regression analyses (Fig. 3 and Supplementary Table 2). The plasma concentration of xanthine showed significant positive correlations with the BMI (R = 0.39, *P* < 0.001), waist circumference (R = 0.42, *P* = 0.001), fasting IRI (R = 0.35, *P* = 0.001), fasting C-peptide (R = 0.32, *P* < 0.01), HOMA-IR (R = 0.27, *P* < 0.01), AST (R = 0.62, *P* < 0.001), ALT (R = 0.56, *P* < 0.001), γ-GTP (R = 0.54, *P* < 0.001), and Fib4-index (R = 0.43, *P* < 0.01), and a negative correlation with the HDL-C (R = − 0.21, *P* < 0.05) in patients with type 2 diabetes.

### Correlations between the plasma concentrations of hypoxanthine and metabolic parameters

**Figure 4:**
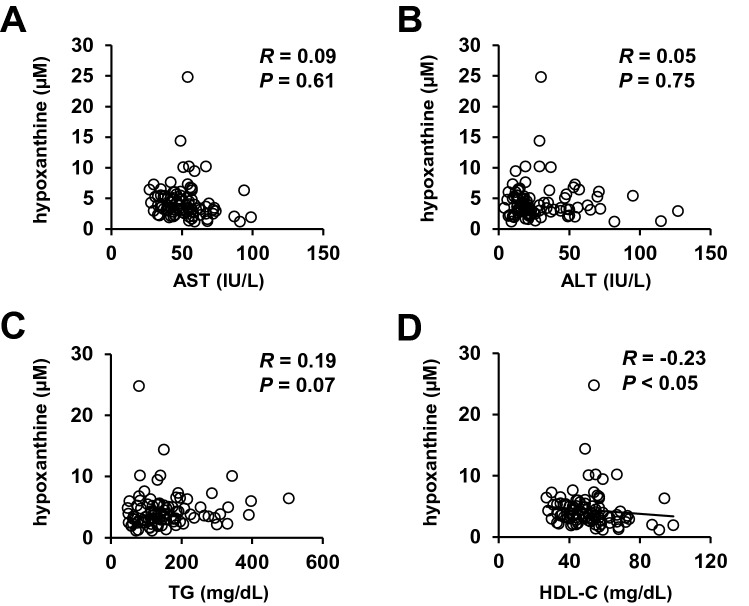
Correlations between the plasma hypoxanthine level and clinical parameters. Correlations between the plasma concentration of plasma hypoxanthine (µM) and serum levels of (**A**) AST (IU/L) and (**B**) ALT (IU/L), (**C**) TG (mg/dL), and (**D**) HDL-C (mg/dL).

We further investigated the correlations between the plasma hypoxanthine levels and clinical parameters in patients with type 2 diabetes (Fig. 4 and Supplementary Table 3). The plasma concentration of hypoxanthine was negatively correlated with the HDL-C (R = − 0.23, *P* < 0.05), but showed no significant correlation with any other parameter.

### Multiple regression analyses to factors independently associated with the plasma XOR activity

Next, we performed stepwise multiple regression analyses to investigate the factors showing independent associations with the plasma XOR activity in patients with type 2 diabetes. In the multiple regression analysis model, the variables of age, sex, HbA1c, serum uric acid, BMI, fasting IRI, HOMA-IR, AST, ALT, and TG were entered as explanatory variables, and the plasma XOR activity was entered as the dependent variable (Table 2). The ALT (β = 0.465, *P* < 0.001), AST (β = 0.365, *P* = 0.003) and TG (β = 0.161, *P* = 0.01) were found to be significantly and independently associated with the plasma XOR activity (Model 1). No association was observed between the serum uric acid (β = 0.034, *P* = 0.807) and the plasma XOR activity in the current study. When ALT and AST were excluded, fasting IRI (β = 0.775, *P* < 0.001) and HOMA-IR (β = − 0.43, *P* = 0.019) showed significant independent associations with the plasma XOR activity (Model 3).

**Table 2:** Multivariate regression analysis to identify factors independently associated with the plasma XOR activity.

	Model 1		Model 2		Model 3	
	Adjusted R^2^		Adjusted R^2^		Adjusted R^2^	
	R^2^ = 0.664		R^2^ = 0.642		R^2^ = 0.200	
	*β*	*p*	*β*	*p*	*β*	*p*
Age	0.076	0.235	0.039	0.568	0.086	0.877
Sex	− 0.073	0.262	− 0.079	0.211	− 0.155	0.104
BMI	− 0.017	0.373	0.036	0.604	0.015	0.904
HbA1c	− 0.055	0.622	− 0.037	0.559	0.012	0.909
UA	0.034	0.807	0.1	0.123	0.099	0.342
Fasting IRI	0.024	0.743	0.096	0.165	0.775	< 0.001
HOMA-IR	− 0.019	0.785	0.047	0.468	− 0.43	0.019
TG	0.161	0.01	–	–	0.037	0.72
AST	0.365	0.003	0.325	0.011	–	–
ALT	0.465	< 0.001	0.509	< 0.001	–	–

Multiple regression analysis to determine factors independently associated with the plasma xanthine oxidase activity. Plasma xanthine oxidase activity was included as a dependent variable, and age, sex, body mass index, HbA1c, uric acid, fasting IRI and HOMA-IR were used as common explanatory variables.

We also included TG, AST and ALT as explanatory variables in model 1, AST and ALT in model 2, and TG in model 3.

### Associations of plasma XOR activity with diabetic vascular complications

**Figure 5:**
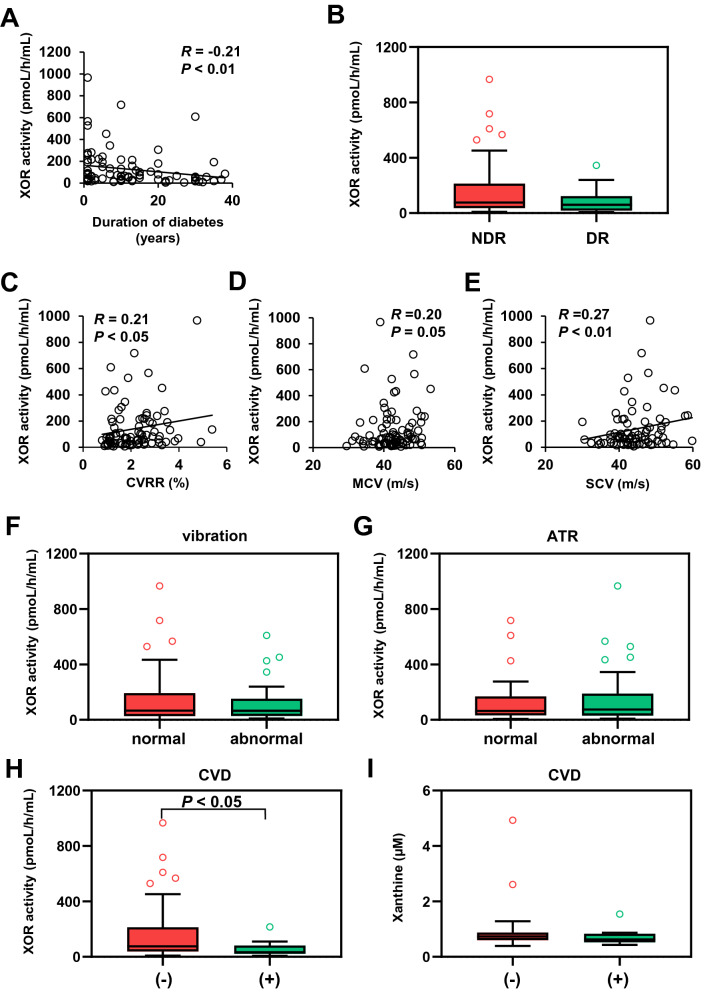
Correlations between plasma XOR activity and diabetic complications. (**A**) Correlation between plasma XOR activity (pmol/h/mL) and the duration of diabetes. (**B**) Plasma XOR activity (pmol/h/mL) in patients with (DR) and without (NDR) diabetic retinopathy. (**C**) Correlation between plasma XOR activity (pmol/h/mL) and coefficient of variation of the R-R interval (CVR-R) (%). (**D**) Correlation between plasma XOR activity (pmol/h/mL) and tibial nerve motor conduction velocity (MCV) (m/s). (**E**) Correlation between plasma XOR activity (pmol/h/mL) and peroneal nerve sensory conduction velocity (SCV) (m/s). (**F**) Plasma XOR activity (pmol/h/mL) in patients with normal and abnormal vibration sense. (**G**) Plasma XOR activity (pmol/h/mL) in patients with normal and blunted Achilles tendon reflex. (**H**) Plasma XOR activity (pmol/h/mL) in patients with or without cardiovascular disease. (**I**) Plasma xanthine concentrations (µM) in patients with and without cardiovascular disease.

Endothelial dysfunction is known to be closely related to the progression of diabetic micro- and macrovascular complications, and atherosclerosis. Since XOR induces endothelial dysfunction via triggering the production of ROS, we next explored the associations between the plasma XOR activity and diabetic vascular complications. In this study, no significant correlations were observed between the plasma XOR activity and parameters of diabetic nephropathy, such as the eGFR or severity of albuminuria. We also focused on the association between the plasma XOR activity and parameters of diabetic neuropathy, such as the coefficient of variation of the R-R interval (CVR-R), tibial motor nerve conduction velocity (MCV) and peroneal sensory nerve conduction velocity (SCV). While the CVR-R (R = 0.21, *P* < 0.05) and SCV (R = 0.27, *P* < 0.01) showed a weak, but significant, correlation with the plasma XOR activity (Fig. 5), the plasma XOR activity did not differ significantly among patients who showed normal, blunted or absent Achilles tendon reflex. No significant difference in the plasma XOR activity was observed between patients with normal and abnormal vibration perception threshold either. The results of several physiological tests for diabetic neuropathy, but not the results of clinical examination for neuropathy, showed the correlations with the plasma XOR activity. In addition, the plasma XOR activity also tended to be decreased in patients with diabetic retinopathy (60.3 pmol/h/mL (18.0–123)) as compared to those without diabetic retinopathy (77.2 pmol/h/mL (36.2–213)), although the difference did not reach statistical significance.

### Plasma XOR activity in patients with cardiovascular disease

We also compared the plasma XOR activity between patients with CVD and patients without CVD. Notably, we found significantly decreased plasma XOR activity levels in patients with CVD (33.3 pmol/h/mL (28.6–78.8)) as compared to patients without CVD (74.5 pmol/h/mL (37.8–204)) (Fig. 5H). In contrast, plasma xanthine concentrations were similar between patients with and without CVD (Fig. 5I). There was no significant correlation between the plasma XOR activity and the brachial-ankle pulse wave velocity (baPWV) or ABI (data not shown). These results indicate that the plasma XOR activity may possibly decrease with the progression of diabetic vascular complications.

## Discussion

Recently, associations between the plasma XOR or XO activity levels and metabolic parameters were reported in patients with metabolic syndrome, renal dysfunction, and cardiovascular disease. The measurement of xanthine and hypoxanthine had been difficult because of leakage from erythrocyte. However, the blood collection using PAXgene Blood DNA tubes (Becton, Dickinson and Company, Japan.) enable the measurements of actual levels of hypoxanthine and xanthine regardless of the time until plasma separation^18^. In the current study, we measured plasma xanthine and hypoxanthine levels using PAXgene Blood DNA tubes and demonstrated associations among the plasma XOR activity, xanthine, and hypoxanthine levels in patients with type 2 diabetes. In addition, we explored whether the plasma XOR activity and/or purine levels might be correlated with the metabolic parameters and risk of vascular complications in patients with type 2 diabetes.

We observed a significant correlation between the plasma XOR activity and plasma xanthine level, but not the plasma hypoxanthine level. This could possibly be attributable to the reutilization of hypoxanthine through the salvage pathway^20^. In the salvage pathway of purine metabolism, which recycles basic materials for reconstitution of DNA, RNA, and purine nucleotides, about 90% of hypoxanthine is reutilized and converted to inosine monophosphate (IMP) by hypoxanthine–guanine phosphoribosyltransferase (HGPRT)^21^. Important roles of HGPRT in cancer or Lesch-Nyhan syndrome are well documented. In addition, although the relevance of HGPRT in the treatment of rheumatic diseases, inflammatory bowel disease, and other pathologies has been reported^22^, the influence of diabetes on the purine salvage pathway has remained obscure. Thus, xanthine and hypoxanthine have different metabolic pathways. In contrast, the plasma xanthine levels were correlated with the plasma hypoxanthine levels. Since hypoxanthine is a precursor of xanthine, this association between hypoxanthine and xanthine might be easy to conceive. Significant associations between the plasma levels of hypoxanthine and xanthine, and between the plasma xanthine and XOR activity were also reported by another study conducted in the general population^23^. The plasma XOR activity and xanthine levels, but not the plasma hypoxanthine level, were correlated with the BMI, unlike the results of a previous study^23^. Hypoxanthine is a known marker of hypoxia^24,25^ and a recent study revealed that hypoxanthine secretion from the adipose tissue increases in response to local hypoxia^26^. It is possible that the production of hypoxanthine from the adipose tissue and other tissues under normal physiological conditions differs from that under the condition of chronic hyperglycemia conditions in type 2 diabetes. The salvage pathway for hypoxanthine metabolism may also possibly have influenced our results, in terms of the correlations with the metabolic parameters.

It is well known that the liver is the main source of XOR, and that hepatic damage caused by an infection and a variety of toxic agents is associated with elevation of the serum XOR activity^27^. Furthermore, many clinical trials have shown correlation between the degree of liver dysfunction and the plasma XOR activity^14,28^. In fact, the plasma XOR activity level was strongly correlated with the serum transaminase and γ-GTP levels in the present study. The plasma xanthine, but not plasma hypoxanthine, level was also correlated with the serum transaminase and γ-GTP levels. Furthermore, the plasma XOR activity tended to show a weak correlation with the Fib4-index, a marker of hepatic fibrosis^29^, although it did not reach statistical significance. However, interestingly, the plasma xanthine concentration showed a significant positive correlation with the Fib4-index. It is unknown whether plasma XOR activity is associated with liver fibrosis. Recently, the contribution of MiR-218-XOR-ROS pathway in the development of non-alcoholic steatohepatitis (NASH) was reported in vitro and in animal model^30^. To the best of our knowledge, this is the first report of the existence of a clinical association between the plasma XOR activity/xanthine levels and liver fibrosis. These results indicate that both the plasma xanthine and plasma XOR activity are possibly associated with the severity of liver fibrosis, as well as the severity of liver dysfunction.

A previous study showed the existence of a relation between the plasma XOR activity and the severity of dyslipidemia in the general population^14^. In this study also, we found a significant positive correlation between the serum triglyceride levels and plasma XOR activity, and a negative correlation between the serum HDL-C levels and plasma XOR activity. Moreover, there were also significant correlations between the plasma xanthine or hypoxanthine and serum HDL-C levels. The plasma XOR activity was correlated with multiple parameters indicative of insulin resistance, such as the fasting IRI, fasting C-peptide, HOMA-IR, and urinary C-peptide levels during the day in the current study. Thus, the plasma XOR activity may be a marker of the severity of metabolic syndrome.

The plasma XOR activity levels of type 2 diabetes patients in the current study was higher than reported levels in Japanese general population^14,23^. In this study, the plasma XOR activity showed no correlation with the fasting glucose level or percent HbA1c in the patients with type 2 diabetes. Previous studies in which patients with type 1 diabetes or subjects from the general population were enrolled, a positive correlation was observed between the plasma XOR activity and the percent HbA1c^31,32^. The association between glycemic control and plasma XOR activity remains controversial. In this study, the percent glycated albumin showed a weak, but significant negative correlation with the plasma XOR activity. In a previous study of hemodialysis patients with type 2 diabetes, the percent glycated albumin showed a positive and independent association with the plasma XOR activity^33^. Our discrepant results could be explained by the fact that the patients in the aforementioned study were on maintenance hemodialysis and the backgrounds of the patients were different from those in our study: (1) the enrolled individuals in our study were hospitalized because of poor glycemic control, and (2) we excluded patients with severe renal dysfunction (eGFR < 30 mL/min/1.73 m^2^), (3) hypoalbuminemia in patients with hemodialysis. Since the percent glycated albumin is a precursor of advanced glycation end products and enhances oxidative stress, it is possible that this result reflected oxidative stress, at least in part. Thus, the association between glycemic control and the plasma XOR activity still remains unclear, and further investigation is required.

The results of our multiple regression analysis identified the serum levels of ALT, AST, and triglyceride as independent indicators of the plasma XOR activity in patients with type 2 diabetes. It is well known that liver dysfunction is associated with increased plasma XOR activity. A previous study also showed associations between the serum levels of liver enzymes/serum triglyceride levels/fasting IRI/HOMA-IR and the plasma XOR activity in the general population^14^. When the serum levels of transaminases were excluded from the possible determinants, fasting IRI and HOMA-IR were found to be independent predictors of the plasma XOR activity. Since any of the serum triglyceride, fasting IRI, or HOMA-IR can reflect insulin resistance, these findings suggest that both liver dysfunction and insulin resistance are associated with the elevation of the plasma XOR activity in patients with type 2 diabetes. Fatty liver, which could be one of the causes of liver dysfunction, is known to be associated with hepatic insulin resistance. A previous study showed that treatment with metformin decreased the serum XOR activity in patients with type 2 diabetes^34^. Our finding of the association between liver fibrosis and the plasma XOR activity also supports the notion of the possible interaction between hepatic insulin resistance and increased plasma XOR activity.

We also found a weak, but significant negative correlation between the plasma XOR activity and the duration of diabetes, indicating that the plasma XOR activity could decrease with the progression of diabetes. This might be consistent with the finding that patients with reduced CVR-R and sensory nerve conduction velocity, which are indicative of advanced diabetic neuropathy, showed decreased plasma XOR activity. However, another study reported that type 2 diabetic patients with diabetic peripheral neuropathy, diagnosed using a bedside scoring system and the Michigan Neuropathy Screening Instrument (MNSI), showed higher plasma XO activity as compared to type 2 diabetic patients with non-diabetic neuropathy^15^. In addition, since there was no significant correlation between the plasma XOR activity and the motor nerve conduction velocity, and patients with impaired vibration perception threshold or blunted Achilles tendon reflex did not show decreased plasma XOR activity, further investigation is needed to elucidate the association between plasma XOR activity and diabetic neuropathy. Patients with diabetic retinopathy also showed a lower XOR activity, although the difference was not statistically significant, as compared to the patients without retinopathy. In the present study, no association was observed between the plasma XOR activity and the presence of diabetic nephropathy. Since we excluded patients with progressive nephropathy from the study and the mean duration of diabetes in the patients enrolled in this study was 10 years, further studies are needed to clarify these relationships.

Notably, patients with CVD showed significantly reduced plasma XOR activity as compared to patients without CVD. The relationship between the risk of cardiovascular events and serum uric acid levels is well known^35^. It has also been reported that the XOR activity levels in tissues and/or the plasma are associated with the incidence of heart failure, cardiovascular events^12,13^. A previous study showed elevated plasma XO activity in patients with the acute coronary events^7^, although the role of XOR in ischemia reperfusion injury is still controversial. In an animal experimental model, inhibition of XOR activity by an XO inhibitor improved the cardiovascular outcomes by reducing superoxide-induced tissue injury^36^. These previous findings certainly indicate the significance of plasma XOR activity as a predictor of cardiovascular events. On the other hand, we observed the decreased plasma XOR activity in patients with CVD, possibly because we analyzed the plasma XOR activity retrospectively in patients with previous cardiovascular events or chronic atherosclerosis. Multiple factors, including chronic hyperglycemia, increased oxidative stress, endothelial dysfunction, and other pathogenetic factors in patients with type 2 diabetes, could also have influenced on our results.

Importantly, circulating XOR binds to the endothelial cells via sulfated glycosaminoglycans on the cell surface, triggering the production of ROS at the vascular endothelium^37^. A shift from the extracellular binding sites to intracellular compartments, by so-called endocytosis, of XOR has also been reported. This phenomenon may explain the decreased plasma XOR activity associated with endothelial dysfunction observed in patients with a long duration of diabetes or with diabetic vascular complications. This notion might also be supported by previous reports of decreased plasma XOR activity in accordance with the severity of chronic kidney disease (CKD)^38,39^. Thus, plasma XOR activity is usually increased in the early stages of diabetes, reflecting insulin resistance or inflammation. In contrast, the levels may decrease with the progression of diabetic vascular complications, known to be associated with severe endothelial dysfunction.

To the best of our knowledge, there have been few previous reports about the relationship between the plasma XOR activity and diabetic vascular complications, these results give a new insight into the pathogenesis of diabetes and associated diseases. Furthermore, our findings might be of practical value if/when XOR inhibitors are used in patients with the expectation of protective effects against oxidative stress; a renoprotective effect of XOR inhibitors has already been reported in patients with CKD.

We demonstrated the significance of the plasma XOR activity and the levels of its substrates, xanthine and hypoxanthine, in patients with type 2 diabetes. Associations have been reported previously between the plasma XOR activity and liver dysfunction/ severity of insulin resistance/the severity of cardiovascular disease. In this study, we revealed, for the first time, the association between the plasma xanthine level and these parameters in patients with diabetes. In addition, the plasma XOR activity was negatively correlated with the duration of diabetes, and the existence of a possible association between the plasma XOR activity and the development of diabetic vascular complications was revealed. Further investigations are needed to determine whether the plasma XOR activity and plasma levels of the related purines could be useful biomarkers of the development of diabetes or its vascular complications.

## Study limitations

There were some limitations of our current study. First, the number of patients in this study was relatively small, and the patients were all Japanese subjects. Second, most of the subjects had poor glycemic control, and further analysis is needed to determine the influence of the grade of glycemic control. Third, the patients were receiving drugs for diabetes, as shown in Table 1; the drugs, including insulin, GLP-1 receptor analogs, metformin, SGLT2 inhibitors and thiazolidinedione, could also influence the serum insulin levels, insulin resistance index, and other parameters. Fourth, we did not include a control group of healthy individuals, because our main objective was to examine the associations between the plasma XOR activity and the risk of diabetic vascular complications and other metabolic disorders in patients with type 2 diabetes. Finally, since this study was a clinical observational, cross-sectional study, we could not determine the causal relations of the plasma XOR activity or purine levels with the other parameters in patients with type 2 diabetes.

## Supplementary Information

Supplementary Information

## Data Availability

The datasets used and analyzed during the current study are available from the corresponding author on reasonable request.
