# Supplementary material for: Association of the plasma xanthine oxidoreductase activity with the metabolic parameters and vascular complications in patients with type 2 diabetes
Source: Sci Rep. 2021 Feb 12;11:3768. doi: 10.1038/s41598-021-83234-9 (PMC7881005; doi:10.1038/s41598-021-83234-9)
Supplement: Supplementary file 1 — Supplementary Information [file 41598_2021_83234_MOESM1_ESM.pdf]

Supplementary Information

Association of the plasma xanthine oxidoreductase activity with the metabolic parameters and vascular complications in patients with type 2 diabetes

Tomoko Okuyama[1], Jun Shirakawa[1,2]*, Takashi Nakamura[3], Takayo Murase[3], Daisuke Miyashita[1], Ryota Inoue[1,2], Mayu Kyohara[1], Yu Togashi[1], Yasuo Terauchi[1]

1. Department of Endocrinology and Metabolism, Graduate School of Medicine, Yokohama City University, Yokohama, Japan
2. Laboratory and Diabetes and Metabolic Disorders. Institute for Molecular and Cellular Regulation (IMCR), Gunma University, Maebashi, Japan
3. Mie Research Laboratories, Sanwa Kagaku Kenkyusho Co., Ltd., Inabe, Mie, Japan.

*Correspondence to: Jun Shirakawa, MD, PhD
Laboratory and Diabetes and Metabolic Disorders
Institute for Molecular and Cellular Regulation (IMCR)
Gunma University3-39-15 Showa-machi, Maebashi 371-8512, Japan
E-mail: jshira-tky@umin.ac.jp
Tel number: +81-27-220-8850, Fax number: +81-27-220-8895

Supplementary Figure 1

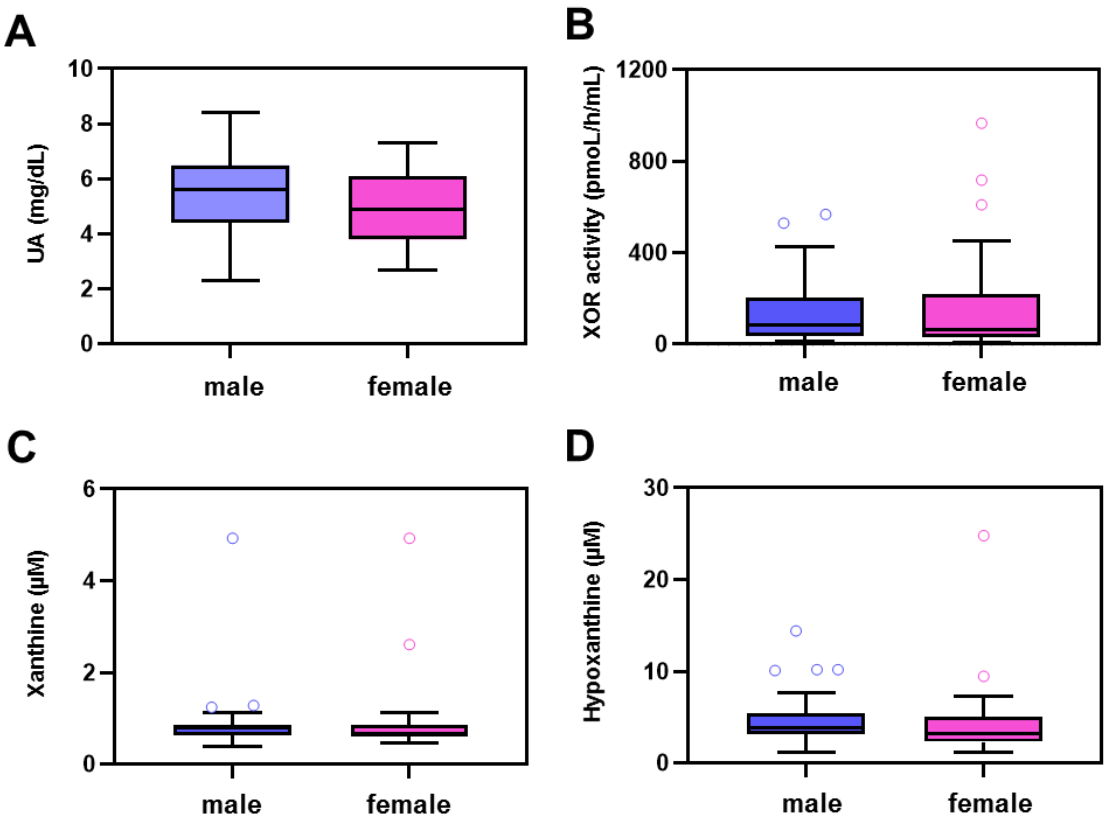

Supplementary Figure 1. Plasma XOR activity, purine catabolism, and uric acid levels in males and females.

(A) serum uric acid levels (mg/dL), (B) plasma XOR activity (pmol/h/mL), (C) plasma xanthine (μM), and (D) plasma hypoxanthine (μM) levels in males and females.

Supplementary Table 1. Correlation analyses for plasma XOR activity.

	R	*P* value
Age	-0.146	0.161
BMI	0.502	0.000
Waist circumference	0.322	0.009
Duration conduction velocity	-0.295	0.006
HbA1c	0.083	0.427
Glycated albumin	-0.266	0.010
Fasting blood glucose	0.002	0.983
Fasting IRI	0.539	0.000
Fasting CPR	0.512	0.000
HOMA-IR	0.466	0.000
eGFR	0.005	0.964
Cystatin C	0.081	0.440
UA	0.417	0.000
LDL-C	0.156	0.134
HDL-C	-0.303	0.003
TG	0.318	0.002
AST	0.808	0.000
ALT	0.881	0.000
γ-GTP	0.759	0.000
Fib4-index	0.310	0.054
ACR	0.037	0.723
Urinary CPR	0.406	0.010

XOR, xanthine oxidoreductase; BMI, body mass index; HbA1c, glycosylated hemoglobin; IRI, immunoreactive insulin; CPR, C-peptide immunoreactivity; HOMA-IR, homeostasis model assessment of insulin resistance; eGFR, estimated glomerular filtration rate; UA, serum uric acid level; T-Chol, serum total cholesterol level; HDL-C, serum high-density lipoprotein cholesterol level; LDL-C, serum low density lipoprotein cholesterol level; TG, serum triglyceride level; AST, serum aspartate aminotransferase level; ALT, serum alanine aminotransferase level; γ-GTP, serum γ-glutamyl transpeptidase level; ACR, urinary albumin to creatinine ratio

Supplementary Table 2. Correlation analyses for plasma xanthine.

	R	*P* value
Age	0.057	0.586
BMI	0.386	0.000
Waist circumference	0.419	0.001
Duration	-0.128	0.239
HbA1c	-0.003	0.975
Glycated albumin	-0.165	0.111
Fasting blood glucose	-0.075	0.472
Fasting IRI	0.347	0.001
Fasting CPR	0.320	0.002
HOMA-IR	0.271	0.009
eGFR	-0.122	0.241
Cystatin C	0.191	0.065
UA	0.392	0.000
LDL-C	0.147	0.157
HDL-C	-0.207	0.045
TG	0.178	0.086
AST	0.620	0.000
ALT	0.562	0.000
γ-GTP	0.535	0.000
Fib4-index	0.427	0.007
ACR	0.124	0.234
Urinary CPR	0.133	0.421

BMI, body mass index; HbA1c, glycosylated hemoglobin; IRI, immunoreactive insulin; CPR, C-peptide immunoreactivity; HOMA-IR, homeostasis model assessment of insulin resistance; eGFR, estimated glomerular filtration rate; UA, serum uric acid level; T-Chol, serum total cholesterol level; HDL-C, serum high-density lipoprotein cholesterol level; LDL-C, serum low density lipoprotein cholesterol level; TG, serum triglyceride level; AST, serum aspartate aminotransferase level; ALT, serum alanine aminotransferase level; γ-GTP, serum γ-glutamyl transpeptidase level; ACR, urinary albumin to creatinine ratio

Supplementary Table 3. Correlation analyses for plasma hypoxanthine.

	R	P value
Age	-0.108	0.301
BMI	0.141	0.176
Waist circumference	0.232	0.063
Duration	-0.144	0.182
HbA1c	0.178	0.086
Glycated albumin	-0.022	0.836
Fasting blood glucose	-0.013	0.898
Fasting IRI	0.089	0.400
Fasting CPR	0.057	0.589
HOMA-IR	0.052	0.623
eGFR	0.104	0.318
Cystatin C	-0.082	0.434
UA	0.046	0.662
LDL-C	0.075	0.471
HDL-C	-0.234	0.023
TG	0.190	0.067
AST	0.085	0.605
ALT	0.053	0.746
γ-GTP	0.107	0.517
Fib4-index	0.007	0.967
ACR	-0.143	0.170
Urinary CPR	0.101	0.543

BMI, body mass index; HbA1c, glycosylated hemoglobin; IRI, immunoreactive insulin; CPR, C-peptide immunoreactivity; HOMA-IR, homeostasis model assessment of insulin resistance; eGFR, estimated glomerular filtration rate; UA, serum uric acid level; T-Chol, serum total cholesterol level; HDL-C, serum high-density lipoprotein cholesterol level; LDL-C, serum low density lipoprotein cholesterol level; TG, serum triglyceride level; AST, serum aspartate aminotransferase level; ALT, serum alanine aminotransferase level; γ-GTP, serum γ-glutamyl transpeptidase level; ACR, urinary albumin to creatinine ratio
